# Dietary carotenoid-rich oil supplementation improves exercise-induced anisocytosis in runners: influences of haptoglobin, MnSOD (Val9Ala), CAT (21A/T) and GPX1 (Pro198Leu) gene polymorphisms in dilutional pseudoanemia (sports anemia)

**DOI:** 10.1590/S1415-47572010005000022

**Published:** 2010-06-01

**Authors:** Ana L. Miranda-Vilela, Arthur K. Akimoto, Penha C. Z. Alves, Luiz C. S. Pereira, Maria N. Klautau-Guimarães, Cesar K. Grisolia

**Affiliations:** 1 Laboratório de Genética, Departamento de Genética e Morfologia, Instituto de Ciências Biológicas, Universidade de Brasília, Brasília, DFBrazil; 2 Laboratórios Sabin, Núcleo de Apoio à Pesquisa, Brasília, DFBrazil

**Keywords:** *Caryocar brasiliense Camb.*, hemogram, hematimetric indexes (MCV, MCH, MCHC and RDW), haptoglobin and antioxidant enzyme polymorphisms

## Abstract

Physical training induces beneficial adaptation, whereas exhaustive exercises increase reactive oxygen-species generation, thereby causing oxidative damage in plasma and erythrocytes, fractions susceptible to lipid peroxidation. Pequi (*Caryocar brasiliense* Camb.) is a Brazilian Cerrado fruit containing a carotenoid-rich oil. The aim was to investigate the effects of pequi-oil on exercise-induced oxidative damage in plasma and erythrocytes, after running in the same environment and undergoing weekly training under the same conditions as to type, intensity and length. Evaluations were accomplished after outdoor running on flat land before and after ingestion of 400 mg pequi-oil capsules for 14 days. Blood samples were taken after running and submitted to TBARS assay and erythrogram analysis. Haptoglobin, MnSOD (Val9Ala), CAT (21A/T) and GPX1 (Pro198Leu) gene polymorphisms were priorly investigated, so as to estimate genetic influence The reduction in erythrocytes, hemoglobin and hematocrit after pequi-oil treatment was notably associated with higher plasma expansion. Except for MCHC (mean corpuscular hemoglobin concentration) and RDW (red cell distribution width), the results were influenced by the polymorphisms studied. The best response to pequi-oil was presented by MnSOD Val/Val, CAT AA or AT genotypes and the GPX1 Pro allele. The significantly lower RDW and higher MHCH values were related to pequi-oil protective effects. Pequi oil, besides possessing other nutritional properties, showed protective blood effects.

## Introduction

Cells continuously produce reactive oxygen species (ROS), mainly as a result of normal oxidative metabolism in the mitochondria (Hermes-Lima, 2004). Under normal circumstances, ROS are neutralized by an elaborate anti-oxidant defence system, consisting of enzymes such as catalase (CAT), superoxide dismutase (SOD), glutathione peroxidase (GPX) and numerous non-enzymatic antioxidants (Hermes-Lima, 2004; Ferreira *et al.*, 2007). Strenuous physical activity increases oxygen consumption and can produce an imbalance between ROS and antioxidants, inducing oxidative stress as a result of increased ROS production (Ji, 1995; Sureda *et al.*, 2005). Acutely, intense exercise is a well accepted model to induce oxidative stress (Alessio, 1993; Ji, 1995), leading to an increase in plasma lipid peroxidation (Clarkson and Thompson, 2000; Ferreira *et al.*, 2007). Exhaustive exercise can induce oxidative damage in blood cells and plasma, which are the most susceptible fractions to suffer from exercise-induced lipid oxidative damage (Sureda *et al.*, 2005).

Exercise results in increased amounts of malondialdehyde (MDA) in the blood, which serve as indirect indicators of lipid peroxidation and can be measured by TBARS assay (Wasowicz et *al*., 1993; Clarkson and Thompson, 2000; Ferreira *et al.*, 2007). Lipid and protein oxidation induced by high-intensity exercise have previously been clearly demonstrated (Alessio, 1993; Ji, 1995); erythrocytes are very sensitive to oxidative stress, because they are unable to repair damaged components, such as proteins, by re-synthesis (nucleus absence), and their membranes are vulnerable to peroxidative damage, besides their poor repair mechanism (Cazzola *et al.*, 2003; Sureda *et al.*, 2005).

On the other hand, a condition referred to as “sports anemia” has been observed in athletes, especially endurance athletes, who presented low-normal hematologic values (red blood cells, hemoglobin, hematocrit) (Carlson and Mawdsley, 1986; Eichner, 1998). However this is a false anemia”, since regular aerobic exercises expand the volume of baseline plasma, thereby diluting red blood cells (RBC) and hemoglobin (Hb) concentration (Eichner, 1998). In other words, the naturally lower hematologic values of an endurance athlete are a dilutional pseudoanemia, which is an adaptation to the acute loss of plasma volume that occurs in initial stages of exercise (Carlson and Mawdsley, 1986; Eichner, 1998). Due to the early reduction in plasma volume, the organism releases renin, aldosterone and vasopressin; as a final result, the baseline plasma volume expands (Carlson and Mawdsley, 1986; Eichner, 1998; Petibois and Déléris, 2005).

The most common true anemia in athletes is iron-deficiency anemia, which occurs mainly in women athletes (Petibois and Déléris, 2005). However, hemolysis can also occur, as a result of mechanical trauma in the capillaries of runners' feet (Carlson and Mawdsley, 1986). In this case, extracellular Hb becomes highly toxic because of the oxidative capacity of iron-containing heme, which participates in the Fenton reaction to produce reactive oxygen species and causes cell injuries (Wassel, 2000; Guéye *et al.*, 2006). Hence, haptoglobin (Hp), a serum glycoprotein, functions as an antioxidant by virtue of its ability to bind to hemoglobin and thereby to prevent Hb-induced oxidative damage (Tseng *et al.*, 2004; Guéye *et al.*, 2006). It has been demonstrated that the protective effect of Hp against this oxidative mechanism is also phenotype dependent (Guéye *et al.*, 2006).

Furthermore, many potentially significant genetic variants related to oxidative stress have already been identified, including single nucleotide polymorphisms (SNPs), Val9Ala in the mitochondrial targeting sequence of the MnSOD gene (NCBI, refSNP ID: rs1799725), 21A/T in the promoter region of the CAT gene (NCBI, refSNP ID: rs7943316), and Pro198Leu of the GPX1 gene (NCBI, refSNP ID: rs1050450), besides others (Mitrunen *et al.*, 2001; Ukkola *et al.*, 2001; Morgenstern, 2004; Zhao *et al.*, 2005). The effect of these variations has not yet been clarified; however, most of the polymorphisms result in changes in the levels or the activities of these enzymes, which can lead to reduction in protection against oxidative stress (Bastaki *et al.*, 2006).

Pequi (*Caryocar brasiliense* Camb.) is a typical tree found in the Brazilian Cerrado, the fruit of which is well known in regional cookery, in popular medicine and for its high nutritional value. Since pequi fruit-pulp-oil contains several carotenoids such as β-carotene, lycopene, ζ-carotene, cryptoflavin, β-cryptoxanthine, anteraxanthine, zeaxanthine, mutatoxanthine, violanxanthine, lutein and neoxanthine (Ramos *et al.*, 2001; Azevedo-Meleiro and Rodriguez-Amaya, 2004; Oliveira *et al.*, 2006; Lima *et al.*, 2007), the aim of this study was to investigate the protective effects of pequi oil against exercise-induced damages in plasma and RBC, after races in the same environment and the same type, intensity and length of weekly training conditions. To estimate if the antioxidant effects of pequi oil were influenced by antioxidant enzyme genotypes, the MnSOD (Val9Ala), CAT (21A/T) and GPX1 (Pro198Leu) genes' polymorphisms were investigated.

## Materials and Methods

###  Study design and participants

The trial was conducted from August 2007 to April 2008, after preclinical and toxicological tests in mice (Miranda-Vilela *et al.*, 2008). Volunteers of both genders (74 men and 45 women) and different age groups (15 to 67) were recruited in high schools, colleges, universities, clubs and companies in Brasília (Federal District). The selection criterion used for the runners was that they had at least a 4,000 m run performance; they were to participate in two races, before (control group) and after (treatment group) ingestion of 400 mg of pequi oil in capsules supplied daily for 14 consecutive days. The choice of this daily ingestion protocol took into account the data from pequi literature and the maximum daily dose of provitamin A carotenoids (25 mg) recommended by the National Agency for Sanitary Surveillance (ANVISA).

The races took place outdoors on flat land, under the same environmental conditions, and the athletes could choose the distance that they would cover (4, 5, 6, 7, 8, 10, 16, 19, 21 km), according to their type, intensity and length of weekly training, guaranteed that it would be the same in the two races. The time needed to finish the races was verified and it was the same in both race, thus guaranteeing the same intensity (time needed to finish the race) before and after pequi oil supplementation. The volunteers were informed about the purpose of the study; all of them received a computer-generated random number and were free to withdraw at any time during the study. After the first race, they received the capsules and were instructed to take them for 14 days during or immediately after lunch until the second race.

The protocol was approved by the Research Ethics Committee for the Health Sciences Faculty of the University of Brasília and by the National Commission for Ethics in Research (CONEP), number 0.001668/2005-18. Written informed consent was obtained from all subjects.

###  Preparation of the capsules

Pequi fruit was obtained *in natura* from the local markets of Brasília/DF (Brazil) and surrounding areas. The internal mesocarp was peeled or grated to obtain the pulp, which was packed in a covered pot and frozen at -86 °C. Pequi pulp-oil was extracted by cold maceration using chloroform as a solvent. The extract was submitted to evaporation under reduced pressure for solvent recovery, and dried at high vacuum. The oil was then incorporated into Aerosil's (colloidal silicon dioxide) q.s.p., guaranteeing ingestion of a daily 400 mg dose. Relative composition is shown in Table 1. The production process was patented as number PI0601631-6 (National Institute of Industrial Property - INPI).

###  Procedures and measurements

Blood samples were drawn with EDTA immediately after races in two rounds: (1) running without pequi-oil supplementation and (2) running after ingestion of 400 mg of pequi oil in capsules supplied daily for 14 consecutive days. Blood samples were used to count RBC and for genotyping of haptoglobin and antioxidant enzymes, while serum samples were submitted to TBARS assay.

###  RBC counting and TBARS assay

RBC counting was carried out in an automated Cell-Dyn 3700 analyzer (Abbott Diagnostics), using internal (Cell-Dyn 22) and external (Program of Excellence for Medical Laboratories - PEML) controls. Values of erythrocytes, hemoglobin, hematocrit and hematimetric indexes (mean cell volume or MCV; mean cell hemoglobin or MCH; mean corpuscular hemoglobin concentration or MCHC; and red cell distribution width or RDW) were estimated and their reference values were listed in Table 2. The TBARS assay was carried out according to Wasowicz *et al.* (1993), and the fluorescence was measured with a Jasco FP-777 spectrofluorometer (excitation: 525 nm, emission: 547 nm).

###  Haptoglobin (Hp) and antioxidant enzymes genotyping

DNA was isolated from the buffy-coat layer using the GFX purification kit (GE Healthcare, Buckinghamshire, England). Hp genotypes were determined by allele-specific PCR (Polymerase Chain Reaction) as described by Yano *et al.* (1998), while Mn-SOD, CAT and GPX1 genotypes were determined by polymerase chain reaction (PCR)-based restriction fragment length polymorphism (RFLP) assays performed as described respectively by Mitrunen *et al.* (2001), Ukkola *et al.* (2001) and Zhao *et al.* (2005). PCR and PCR-based RFLP products were resolved in non-denaturing polyacrylamide gels stained with silver nitrate.

###  Statistical analysis

To reveal contingent differences between the groups involved, statistical significance was assessed by T-test for dependent samples. Statistical analysis was carried out using SPSS (Statistical Package for the Social Sciences) version 17.0 and SAS (Statistical Analysis System) version 9.1.3, service pack 3. Data were expressed as mean ± SE (standard error of mean) and values of p < 0.05 were considered statistically significant. The statistical factors analyzed were total and gender groups, age (15-19, 20-24, 25-29, 30-34, 35-39, 40-44 and 45 year-olds up) and distance covered (4-5, 6-7, 8-10 and 16-21 km).

The influence of Hp, MnSOD, CAT and GPX1 genotypes (genetic markers) was investigated through the following ANOVA model: *Y*_*ijk*_ = μ + τ_*i*_ + ρ_*j*_ + (τρ)_*ij*_ + *e*_*ijk*_, where μ is the general mean, τ_*i*_ the pequi-oil effect, ρ_*j*_ the effect of the analyzed variable and *e*_*ijk*_ the random error, which must present normal distribution with mean zero and constant variance. The Fisher LSD test was used to verify differences between the variable categories (genotypes).

Allelic and genotypic frequencies were estimated by gene counting andthe goodness of fit of the genotype distribution for to Hardy-Weinberg equilibrium (HWE) was tested by chi-square (χ^2^ test). Values of p > 0.05 were considered in HWE and were generated by Genepopweb statistical program version 3.4.

## Results

###  RBC counting

After supplementation, there was a general downward trend for erythrocytes, hemoglobin, hematocrit and anisocytosis (RDW values), accompanied by an increase in MCV, MCH and MCHC values; all of them were inside the reference values (Table 3). Results of the total group were significant for erythrocytes (p = 0.0007), hematocrit (p = 0.0011) and RDW (p = 0.0000), which decreased, as well as for MCH (p = 0.0001) and MCHC (p = 0.0002), which increased. For women, there was a significant decrease in erythrocytes (p = 0.0453) and hematocrit (p = 0.0305) and a significant increase in MCH (p = 0.0116) and MCHC (p = 0.0081). Men presented a significant decline in erythrocytes (p = 0.0068), hematocrit (p = 0.0246) and RDW (p = 0.0000), and a significant enhancement of MCH (p = 0.0066) and MCHC (p = 0.0316). Significant RDW reductions were observed in age groups of 15-19, 35-39 and 40-44 years. The second group presented also a significant increase in the MCH (p = 0.0084) and MCHC (p = 0.0379) values after pequi-oil treatment (Table 4). The significant drop in erythrocyte and hematocrit values, although observed for the group as a whole, was particularly related to the age groups 15-19 and 25-29, and distances of 4-5 and 8-10 km. For these distances, there was also a significant increase in MCH (p = 0.0467 and p = 0.0003, respectively) and MCHC (p = 0.0308 and p = 0.0011, respectively) values. RDW values decreased significantly at almost all distances after supplementation, except for 16-21 km (Table 5).

**Figure 1:**
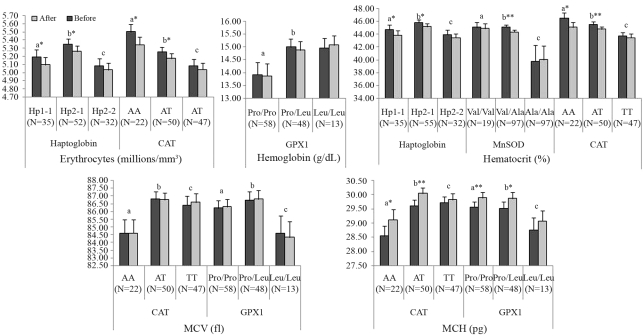
Influence of Haptoglobin (Hp), MnSOD (Val9Ala), CAT (-21A/T) and GPX1 (Pro198Leu) gene polymorphisms on erythrogram results before and after pequi-oil supplementation.Data are expressed as mean ± SE (standard error of mean). Asterisks indicate significant (*p < 0.05) and highly significant (**p < 0.01) differences in the comparison of before-after values by the T-test for dependent samples. The alphabet letters indicate significant differences detected by Fisher's LSD test between the variable's categories (genotypes), as follow: Erythrocytes - for Hp a = significant compared to b (p = 0.03626), b = significant compared to c (p = 0.0015); for CAT a = significant compared to b (p = 0.0176) and c (p < 0.0001), b = significant compared to c (p = 0.0030); Hemoglobin - for GPX1 a = significant compared to b (p = 0.0341); Hematocrit - for Hp a = significant compared to b (p = 0.0276), b = significant compared to c (p = 0.0020); for MnSOD a = significant compared to c (p = 0.0016), b = significant compared to c (p = 0.0015); for CAT a = significant compared to c (p = 0.0009), b = significant compared to c (p = 0.0030); MCV (mean cell volume) - for CAT a = significant compared to b (p = 0.0015) and c (p = 0.0061); for GPX1 a = significant compared to c (p = 0.0285), b = significant compared to c (p = 0.0061); and MCH (mean cell hemoglobin) - for CAT a = significant compared to b (p = 0.0003) and c (p = 0.0006); for GPX1 a = significant compared to c (p = 0.0134), b = significant compared to c (p = 0.0206).

The ANOVA model indicated that results of erythrocytes were influenced by Hp (p = 0.0040) and CAT (p = 0.0003) polymorphisms; hemoglobin by GPX1 (p = 0.0234) polymorphism, hematocrit by Hp (p = 0.0048), MnSOD (p = 0.0053) and CAT (p = 0.0009) polymorphisms; MCV by CAT (p = 0.0047) and GPX1 (p = 0.0230) polymorphisms; and MCH by CAT (p = 0,0006) and GPX1 (p = 0.0402) polymorphisms (Figure 1). Concerning MCHC and RDW values, the ANOVA model indicated that these hematimetric indexes were influenced only by the pequi oil treatment; that is, the MCHC and RDW values were not influenced by the gene's polymorphism.

###  TBARS assay

**Figure 2:**
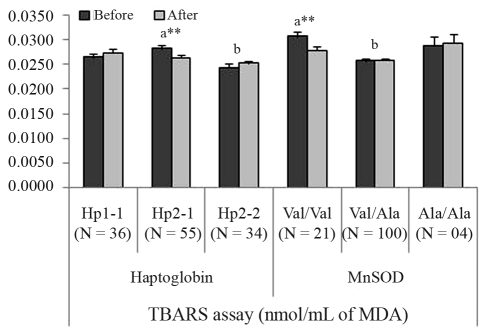
Influence of Haptoglobin (Hp) and MnSOD (Val9Ala) gene polymorphisms on the results of TBARS assay before and after pequi-oil supplementation. Data are expressed as mean ± SE (standard error of mean). Asterisks indicate highly significant (**p < 0.01) differences in the comparison of before-after values by the T-test for dependent samples. Letters indicate significant differences detected by Fisher's LSD test between the variable's categories (genotypes): for Hp a = significant compared to b (p = 0.0185); for MnSOD a = significant compared to b (p = 0.0032).

After pequi-oil supplementation, there was a non-significant drop in those values specifically related to men, with no effect being observed in women. Significant values appeared for MDA increased at 4-5 km (p = 0.0427) and for MDA decreased at 6-7 km (p = 0.0034); the results were influenced by Hp (p = 0.0008) and MnSOD (p = 0.0072) polymorphisms (Figure 2).

###  Genetic marker analysis

Table 6 summarizes the distribution of Haptoglobin, MnSOD, CAT and GPX1 allele frequencies, the genetic diversity parameters, the genotype frequencies and the Hardy-Weinberg equilibrium (HWE) data for chi-square (χ^2^) test. Results indicated a significant deviation from Hardy-Weinberg equilibrium (HWE) for the MnSOD locus (p = 0.0000), with a heterozygote excess (p = 0.0000). MnSOD locus presented a heterozygosity-observed (Ho) value higher than the heterozygosity-expected (He) value, and an FIS (inbreeding coefficient) value (FIS = 0.6302) compatible with selection in favour of heterozygotes. The genotypic distributions of Hp, CAT and GPX1 loci were in accordance with HWE.

###  Adverse effects

No subjects withdrew from the study because of discomfort or adverse effects associated with the treatment. Eleven subjects experienced heavy drowsiness, while four reported insomnia; six volunteers had a mild intestinal disorder, two constipation, and three increased flatulence, one man complained of heartburn, two women reported increased acne and one woman described the appearance of painful subcutaneous nodules on the arms. All these symptoms were noticed within the first 3 to 4 days of treatment, disappearing soon afterwards.

## Discussion

Athletes, especially distance runners, tend to develop a condition known as sports anemia or pseudoanemia, which provokes temporary reduction in hematologic values (red blood cells, hemoglobin and hematocrit) as a result of plasma volume expansion (Carlson and Mawdsley, 1986; Eichner, 1998). Such transitory reductions are a beneficial physiological adaptation to aerobic exercises (Carlson and Mawdsley, 1986; Eichner, 1998; Petibois and Déléris, 2005), since they decrease the resistance of blood flow, improve sweating and increase ejection volume of the heart (Eichner, 1998). Since the naturally lower hematologic values of endurance athletes is a dilutional pseudoanemia, the “footstrike” hemolysis that occurs as a result of mechanical trauma in the capillaries of runners' feet is unlikely to cause anemia (Carlson and Mawdsley, 1986; Eichner, 1998). Therefore, the most common true anemia in athletes is due to iron-deficiency, which occurs mainly in women (Eichner, 1998). By sweating, athletes generally lose amounts of iron, which should be supplied by the diet. The principal cause of iron-deficiency in athletic women is insufficient intake of iron in their diet to supply their greater physiological necessities, since they also lose iron during menstruation (Eichner, 1998).

In this context, the general downward trend for erythrocytes, hemoglobin and hematocrit after pequi-oil supplementation was mainly related to plasma expansion rather than to hemolysis, since this drop was accompanied by significantly lower RDW and higher MCH and MCHC. Because RDW is the most commonly reported index of the variation or degree of anisocytosis in cell volume within the red cell population (Zago *et al.*, 2004), and erythrocytes are prone to suffer from exercise-induced lipid oxidative damage (Sureda *et al.*, 2005), mechanical stress, and cytosolic and extracellular pH changes (Petibois and Déléris, 2005), the significant reduction in RDW values indicates that pequi oil exerted protective effects on RBC. Moreover, after pequi-oil supplementation, RDW values returned to their normal reference values. In addition, while MCH indicates the average amount of oxygen-carrying hemoglobin inside an RBC, MCHC indicates the mean concentration of hemoglobin inside red cells (in respect to 1 dL of erythrocytes) (Zago *et al.*, 2004). Both increased after supplementation. Thus, these results suggest that pequi oil improved the blood oxygen-carrying capacity. However, except for MCHC and RDW values, results were influenced by the polymorphisms studied.

The Hp phenotypes determine the serum levels of the Hp-glycoprotein, and they differ in plasma concentration of Hp and in antioxidant activities, besides others, presenting different clinical consequences (Yano *et al.*, 1998; Wassel, 2000; Tseng *et al.*, 2004). Since various functional properties of Hp phenotypes have been described as a direct consequence of protection against oxidative stress (Wassel, 2000; Guéye *et al.*, 2006), our results suggest that the higher Hp2-1 genotype frequency can be associated with an improved capacity to carry oxygen, although intermediate antioxidant activities between Hp1-1 and Hp2-212 have been cited. Given that Hp2-2 presented the lowest MDA values in TBARS assay, its lower frequency in the athlete samples could be related to a lower degree of aerobic capacity in relation to the other genotypes.

MnSOD (EC 1.15.1.1, MIM *147460) is a mitochondrial enzyme that catalyzes the dismutation reaction of superoxide radicals (O2^•-^) to hydrogen peroxide (H_2_O_2_) (Hermes-Lima, 2004). It is encoded by a nuclear gene located on chromosome 6q25.3 (Bastaki *et al.*, 2006). The enzyme is synthesized with a mitochondrial targeting sequence (MTS), which drives its mitochondrial import. In the mitochondrial matrix, the MTS is cleaved, and the mature protein assembles into the active tetramer (Akyol *et al.*, 2005). The valine to alanine substitution in MnSOD MTS induces a conformational change from an α-helix to a β-sheet, which has been reported to change mitochondrial processing efficiency, affect the transport of MnSOD to the mitochondria, and to decrease MnSOD efficiency against oxidative stress (Akyol *et al.*, 2005). Given that the present work was carried out with athletes and MnSOD is under selective pressure, our results suggest that MnSOD heterozygosis favours defence against oxidative stress. The Val/Ala genotype presented the lowest lipid peroxidation levels, whereas MnSOD Val/Val genotypes responded better to pequi-oil supplementation.

Catalase (CAT, E.C.1.11.1.6, MIM +115500) is the main regulator of hydrogen peroxide metabolism and its high concentrations in erythrocytes provides a defence against high concentrations of hydrogen peroxide (Góth *et al.*, 2004; Hermes-Lima, 2004; Sureda *et al.*, 2005; Ferreira *et al.*, 2007). Although CAT 21A/T polymorphism has not been associated with any alteration in catalase activity (Góth *et al.*, 2004), individuals carrying a variant T allele presented higher MCH values, suggesting superior efficiency in oxygen transport. However, the best response to pequi-oil treatment was presented by AA and AT genotypes. These genotypes showed a significant decrease in erythrocytes and hematocrit values after supplementation, but they also presented a significant increase in MCH values, confirming the previous suggestion that the general downward trend for erythrocytes, hemoglobin and hematocrit after pequi oil was mainly related to plasma expansion.

The antioxidant enzyme glutathione peroxidase 1 (GPX1, EC 1.11.1.9, MIM +138320) is part of the enzymatic antioxidant defence preventing oxidative damage to biomolecules, by detoxifying hydrogen peroxide and organic hydroperoxides using glutathione in its reduced form (GSH) as a co-substrate (Hermes-Lima, 2004; Sureda *et al.*, 2005; Zhao *et al.*, 2005; Ferreira *et al.*, 2007). The GPX1 gene encoding the isoenzyme particularly abundant in erythrocytes (Sureda *et al.*, 2005; Zhao *et al.*, 2005) and some studies have indicated that a variant Leu allele affects the GPX1 activity, which becomes less responsive to stimulation. The increased risk of some kinds of cancer has also been associated with variant Leu allele. However, such associations have not been consistently observed in all populations, since Leu allele frequency varies by ethnic group (Zhao *et al.*, 2005).

In the present study, although hemoglobin concentration in the Pro/Pro genotype proved to be significantly lower than in the Pro/Leu, MCH values were similar to heterozygous Pro/Leu and these values were significantly higher than those shown by Leu/Leu individuals. Moreover, only individuals carrying the Pro allele presented a significant increase in MCH values after pequi-oil treatment. These results suggest that subjects carrying the Pro allele may present a higher efficiency of each cell in oxygen transport, besides having a better response to antioxidant supplementation.

It is important to note that the antioxidant system, working in parallel with exercise training, is a known inducer of antioxidant enzymes. However, although regular exercise causes adaptations, which reduce the incidence of oxidative stress, increased antioxidant enzyme activities are insufficient to completely eliminate the ROS produced after intense exercise (Sureda *et al.*, 2005; Ferreira *et al.*, 2007). Since the elite athletes who participated in this study covered distances of 6 km and more, influence of the distance covered is expected in the results of TBARS assay, as well as a higher lipidic peroxidation for 4-5 km runners. Nevertheless, after pequi-oil treatment, there was also a significant increase in MCH and MCHC values (within the reference values) at this distance, thereby corroborating the previous suggestion that pequi oil aided in improving blood oxygen-carrying capacity. Although the significant drop in MDA values (TBARS assay) appeared only at the 6-7 km mark after supplementation, there was also a decrease in MDA values at the 8-10 and 16-21 km marks, thus indicating that pequi oil was biologically efficient in reducing lipid peroxidation in trained athletes.

Moreover, it has been suggested that when trained athletes run a comparatively short distance, alterations in erythrocyte antioxidant status may occur even though plasma indices of lipid peroxidation are unaffected (Duthie *et al.*, 1990). Our results are in accordance with this suggestion; they also indicate differences in response that depend on gender, since after pequi-oil supplementation no effect was observed in lipid peroxidation among females, despite significant improvements in RBC parameters. However, except for MCHC and RDW values, results were influenced by the polymorphisms studied.

In summary, our results suggest that pequi oil aided in improving both exercise-induced anisocytosis in runners as well as blood oxygen-carrying capacity. However, except for MCHC and RDW values, the results were influenced by the genetic background. The best response to pequi-oil treatment was presented by subjects carrying MnSOD Val/Val genotype, CAT AA or AT genotype and GPX1 Pro allele. Therefore, pequi oil, as well as possessing many other nutritional properties, is a good candidate for use as an antioxidant supplement.

## Figures and Tables

**Table 1:** Relative composition of pequi (*Caryocar brasiliense* Camb.) pulp-oil capsules. Omega (ω), which is defined according to carbon numeration associated with the first double bonds (3rd, 6th, 7th or 9th) from the methyl group, is correlated to unsaturated fatty acids.

Fatty acids^1^		
Saturated	Carbon number	Quantity (% per 100 g of pequi pulp fruit)
Palmitic	C16:0	41.78
Stearic	C18:0	1.28
Araquidic	C20:0	0.12
	Total	43.18

Unsaturated		
Mono-unsaturated		
Oleic	C18:1 (ω9)	54.28
Palmitoleic	C16:1 (ω7)	0.67
Bi-unsaturated		
Linoleic	C18:2 (ω6)	1.36
Tri-unsaturated		
Linolenic	C18:3 (ω3)	0.51
	Total	56.82

Carotenoids^2^		Quantity (mg/100 g of pequi pulp fruit)

Provitamin A		6.26-11.5
Lycopene		1.12-2.08
	Total	6.75-28.66

^1^Miranda-Vilela *et al.*, 2009 and present study. ^2^Ramo*s et al*., 2001; Azevedo-Meleiro and Rodriguez-Amaya, 2004; Oliveir*a et al*., 2006; Lim*a et al*., 2007.

**Table 2:** Reference values of RBC and hematimetric index

Hemogram	Reference values	
	Men	Women
Erythrocytes (millions/mm^3^)	4.50 to 6.10	4.00 to 5.40
Hemoglobin (g/dL)	13.0 to 16.5	12.0 to 15.8
Hematocrit (%)	36.0 to 54.0	33.0 to 47.8
MCV (fl = femtoliters)	80.0 to 98.0	80.0 to 98.0
MCH (pg = picograms)	26.8 to 32.9	26.2 to 32.6
MCHC (g/% or g/dL)	32.0 to 36.0	32.0 to 36.0
RDW (%)	12.0 to 14.5	12.0 to 14.5

g/dL = grams per deciliter; g/% = grams per percentage.

**Table 3:** Influence of pequi-oil supplementation on the results from the erythrogram of total, men and women groups.

	Erythrocytes (millions/mm^3^)			Hemoglobin (g/dL)			Hematocrit (%)			MCV (fl)			MCH (pg)			MCHC (g/% ou g/dL)			RDW (%)	
	Before	After		Before	After		Before	After		Before	After		Before	After		Before	After		Before	After
Total (N = 119)	5.23 ± 0.05	5.15 ± 0.05**		14.47 ± 0.27	14.41 ± 0.28		45.01 ± 0.34	44.33 ± 0.33**		86.25 ± 0.35	86.29 ± 0.35		29.46 ± 0.14	29.79 ± 0.14**		34.16 ± 0.09	34.53 ± 0.06**		14.84 ± 0.09	14.26 ± 0.10**
Men (N = 74)	5.47 ± 0.05	5.40 ± 0.05**		14.83 ± 0.39	14.77 ± 0.39		46.85 ± 0.33	46.25 ± 0.31*		85.79 ± 0.46	85.93 ± 0.48		29.33 ± 0.19	29.61 ± 0.19**		34.18 ± 0.11	34.47 ± 0.08*		14.96 ± 0.12	14.18 ± 0.11**
Women (N = 45)	4.83 ± 0.05	4.75 ± 0.05*		13.88 ± 0.33	13.81 ± 0.33		41.98 ± 0.46	41.17 ± 0.42*		86.99 ± 0.49	86.89 ± 0.47		29.68 ± 0.21	30.08 ± 0.19*		34.12 ± 0.16	34.63 ± 0.09**		14.65 ± 0.14	14.38 ± 0.18

Data are expressed as mean ± SE (standard error of mean). MCV = mean cell volume; MCH = mean cell hemoglobin; MCHC = mean corpuscular hemoglobin concentration; RDW = Red Cell Distribution Width; g/dL = grams per deciliter; fl = femtoliters; pg = picograms; g/% = grams per percentage. Asterisks indicate significant (*p < 0.05) and highly significant (**p < 0.01) differences in the comparison of before-after values by the T-test for dependent samples. N = sample size.

**Table 4:** Influence of pequi oil supplementation on RBC and hematimetric indices in the age groups.

Age groups	Erythrocytes (millions/mm^3^)			Hemoglobin (g/dL)			Hematocrit (%)			MCV (fl)			MCH (pg)			MCHC (g/% ou g/dL)			RDW (%)	
	Before	After		Before	After		Before	After		Before	After		Before	After		Before	After		Before	After
15-19 (N = 20)	5.35 ± 0.12	5.17 ± 0.13**		15.50 ± 0.27	15.07 ± 0.29*		45.35 ± 0.83	43.71 ± 0.88**		84.92 ± 0.80	84.84 ± 0.78		29.05 ± 0.34	29.28 ± 0.33		34.18 ± 0.16	34.51 ± 0.13		14.25 ± 0.19	14.26 ± 0.24
20-24 (N = 24)	5.26 ± 0.10	5.22 ± 0.10		14.10 ± 0.74	14.15 ± 0.76		45.24 ± 0.77	44.96 ± 0.81		86.16 ± 0.82	86.23 ± 0.81		29.45 ± 0.32	29.82 ± 0.35		34.20 ± 0.20	34.58 ± 0.15		14.86 ± 0.21	14.24 ± 0.23**
25-29 (N = 23)	5.35 ± 0.11	5.24 ± 0.11*		12.76 ± 0.92	12.69 ± 0.95		45.97 ± 0.80	44.91 ± 0.75*		86.42 ± 0.92	86.32 ± 0.94		29.27 ± 0.34	29.66 ± 0.32		33.88 ± 0.24	34.37 ± 0.13		14.99 ± 0.21	14.02 ± 0.16**
30-34 (N = 12)	5.22 ± 0.16	5.16 ± 0.14		14.59 ± 0.97	14.55 ± 0.94		44.96 ± 1.20	44.74 ± 1.02		86.31 ± 0.84	86.87 ± 0.95		29.54 ± 0.38	29.87 ± 0.44		34.22 ± 0.29	34.38 ± 0.24		14.77 ± 0.17	13.91 ± 0.25*
35-39 (N = 15)	5.26 ± 0.13	5.20 ± 0.12		15.07 ± 0.36	15.17 ± 0.35		44.37 ± 1.04	43.99 ± 1.03		84.43 ± 1.00	84.59 ± 1.03		28.67 ± 0.41	29.20 ± 0.34**		33.96 ± 0.30	34.53 ± 0.16*		15.23 ± 0.35	14.77 ± 0.42
40-44 (N = 09)	5.08 ± 0.11	5.05 ± 0.09		15.19 ± 0.33	15.30 ± 0.29		44.49 ± 1.01	44.06 ± 0.79		87.59 ± 0.70	87.29 ± 0.70		29.93 ± 0.37	30.32 ± 0.30		34.18 ± 0.33	34.74 ± 0.18		14.60 ± 0.29	14.01 ± 0.29*
≥ 45 (N = 16)	4.94 ± 0.09	4.90 ± 0.09		15.16 ± 0.36	15.11 ± 0.34		43.79 ± 0.93	43.50 ± 0.90		88.68 ± 0.65	88.76 ± 0.68		30.68 ± 0.31	30.79 ± 0.32		34.60 ± 0.21	34.70 ± 0.17		15.18 ± 0.18	14.54 ± 0.18*

Data are expressed as mean ± SE (standard error of mean). MCV = mean cell volume; MCH = mean cell hemoglobin; MCHC = mean corpuscular hemoglobin concentration; RDW = Red Cell Distribution Width; g/dL = grams per deciliter; fl = femtoliters; pg = picograms; g/% = grams per percentage. Asterisks indicate significant (*p < 0.05) and highly significant (**p < 0.01) differences in the comparison of before-after values by the T-test for dependent samples. N = sample size.

**Table 5:** Influence of pequi oil supplementation on RBC and hematimetric indices at the different distances covered (km)

km	Erythrocytes (millions/mm^3^)			Hemoglobin (g/dL)			Hematocrit (%)			MCV (fl)			MCH (pg)			MCHC (g/% ou g/dL)			RDW (%)	
	Before	After		Before	After		Before	After		Before	After		Before	After		Before	After		Before	After
4-5 (N = 49)	5.32 ± 0.08	5.21 ± 0.08**		13.81 ± 0.56	13.66 ± 0.57		45.53 ± 0.57	44.55 ± 0.56**		85.74 ± 0.61	85.76 ± 0.60		29.30 ± 0.24	29.59 ± 0.25*		34.16 ± 0.13	34.50 ± 0.09*		14.53 ± 0.14	13.98 ± 0.13**
6-7 (N = 33)	5.12 ± 0.07	5.08 ± 0.07		15.10 ± 0.19	15.10 ± 0.20		44.28 ± 0.50	44.09 ± 0.58		86.84 ± 0.48	87.04 ± 0.52		29.58 ± 0.18	29.82 ± 0.19		34.10 ± 0.19	34.26 ± 0.11		14.80 ± 0.15	14.20 ± 0.18**
8-10 (N = 30)	5.25 ± 0.10	5.15 ± 0.08**		14.79 ± 0.52	14.74 ± 0.50		45.42 ± 0.74	44.49 ± 0.62*		86.68 ± 0.62	86.53 ± 0.63		29.62 ± 0.29	30.15 ± 0.25**		34.17 ± 0.20	34.84 ± 0.09**		15.04 ± 0.16	14.44 ± 0.17**
16-21 (N = 07)	5.05 ± 0.13	5.07 ± 0.18		14.81 ± 0.58	14.97 ± 0.58		43.03 ± 1.58	43.26 ± 1.77		85.09 ± 2.11	85.41 ± 2.14*		29.30 ± 0.87	29.56 ± 0.76		34.40 ± 0.33	34.64 ± 0.26		16.34 ± 0.38	15.69 ± 0.64

Data are expressed as mean ± SE (standard error of mean). MCV = mean cell volume; MCH = mean cell hemoglobin; MCHC = mean corpuscular hemoglobin concentration; RDW = Red Cell Distribution Width; g/dL = grams per deciliter; fl = femtoliters; pg = picograms; g/% = grams per percentage. Asterisks indicate significant (*p < 0.05) and highly significant (**p < 0.01) differences in the comparison of before-after values by the T-test for dependent samples. N = sample size.

**Table 6:** Distribution of Haptoglobin, MnSOD, CAT and GPX1 allele frequencies, genetic diversity parameters, genotype frequencies and Hardy-Weinberg equilibrium (HWE) data for chi-square (χ^2^) test.

Alleles	Allele frequencies	Heterozygosity-observed (H_o_)	Heterozygosity-expected (H_e_)	Genotypes	Genotype frequencies	Number of observed individuals	Number of expected individuals	HWE test (p-values)
Haptoglobin				Haptoglobin				
Hp^*1^	0.508	0.504	0.617	1-1	0.288	36	32.26	
Hp^*2^	0.492	0.504	0.617	2-1	0.440	55	62.49	0.2181
				2-2	0.272	34	30.26	

MnSOD				MnSOD				
Val	0.568	0.800	0.491	Val/Val	0.168	21	40.33	
Ala	0.432	0.800	0.491	Val/Ala	0.800	100	61.34	0.0000
				Ala/Ala	0.032	04	23.33	

CAT				CAT				
A	0.396	0.424	0.478	AA	0.184	23	19.60	
T	0.604	0.424	0.478	AT	0.424	53	59.80	0.1944
				TT	0.392	49	45.60	

GPX1				GPX1				
Pro	0.688	0.400	0.429	Pro/Pro	0.488	61	59.17	
Leu	0.312	0.400	0.429	Pro/Leu	0.400	50	53.66	0.5374
				Leu/Leu	0.112	14	12.17	

p-values data for heterozygote excess (*p = 0.0000) were generated using the statistical program Genepopweb version 3.4.
